# Supplementary material for: High-throughput single nucleus total RNA sequencing of formalin-fixed paraffin-embedded tissues by snRandom-seq
Source: Nat Commun. 2023 May 12;14:2734. doi: 10.1038/s41467-023-38409-5 (PMC10182092; doi:10.1038/s41467-023-38409-5)
Supplement: Supplementary file 1 — Supplementary Information [file 41467_2023_38409_MOESM1_ESM.pdf]

Supplementary Information

High-throughput single nucleus total RNA sequencing of formalin-fixed paraffin-embedded tissues by snRandom-seq

Ziye Xu[1,2#], Tianyu Zhang[3#], Hongyu Chen[4,5,6#], Yuyi Zhu[2], Yuexiao Lv[2], Shunji Zhang[7], Jiaye Chen[8], Haide Chen[2], Lili Yang[9], Weiqin Jiang[10], Shengyu Ni[3], Fangru Lu[3], Zhaolun Wang[3], Hao Yang[3], Ling Dong[3], Feng Chen[9], Hong Zhang[7,11], Yu Chen[1], Jiong Liu[3], Dandan Zhang[12,13], Longjiang Fan[4,5,6*], Guoji Guo[2*], Yongcheng Wang[1,2,7*]

[1]Department of Laboratory Medicine, the First Affiliated Hospital, Zhejiang University School of Medicine, Hangzhou, China

[2]Liangzhu Laboratory, Zhejiang University Medical Center, Hangzhou, China

[3]M20 Genomics, Hangzhou, China

[4]School of Medicine, Hangzhou City University, Hangzhou, China

[5]Institute of Bioinformatics, Zhejiang University, Hangzhou, China

[6]James D. Watson Institute of Genome Sciences, Zhejiang University, Hangzhou, China

[7]College of Biomedical Engineering and Instrument Science, Zhejiang University, Hangzhou, China

[8]Department of Biomedical Informatics, Harvard Medical School, Boston, United States

[9]Department of Radiology, the First Affiliated Hospital, Zhejiang University School of Medicine, Hangzhou, China.

[10]Departments of Colorectal Surgery, the First Affiliated Hospital, Zhejiang University, Hangzhou, China

[11]Department of Nuclear Medicine and PET/CT Center, the Second Affiliated Hospital, Zhejiang University School of Medicine, Hangzhou, China

[12]Department of Pathology, and Department of Medical Oncology of the Second Affiliated Hospital,

Zhejiang University School of Medicine, Hangzhou, Zhejiang, China

[13]Department of Pathology, Key Laboratory of Disease Proteomics of Zhejiang Province, School of Medicine, Zhejiang University, Hangzhou, China

[#] These authors contributed equally to this work.

*Corresponding Author: Yongcheng Wang (yongcheng@zju.edu.cn), Guoji Guo(ggj@zju.edu.cn), and Longjiang Fan (fanlj@zju.edu.cn).

Supplementary Figures

a

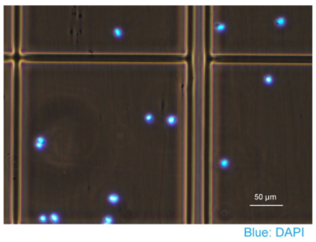

b

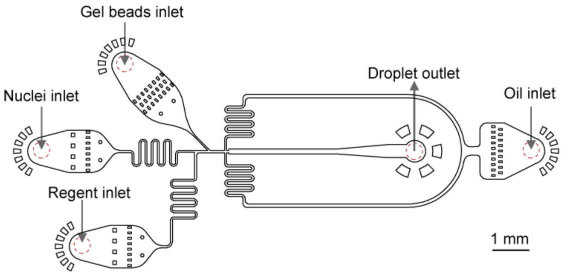

c

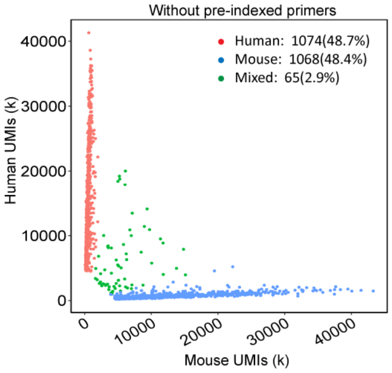

d

Biotype	Count
protein coding	16744
lncRNA	9590
processed pseudogene	1436
unprocessed pseudogene	529
transcribed unprocessed pseudogene	496
snRNA	236
TEC	208
rRNA pseudogene	173
transcribed processed pseudogene	173
misc RNA	163
transcribed unitary pseudogene	99
miRNA	36
unitary pseudogene	30
Mt tRNA	22
snoRNA	21
polymorphic pseudogene	15
rRNA	13
TR V gene	8
pseudogene	3
IG C gene	2
IG V gene	2
IG V pseudogene	2
Mt rRNA	2
TR C gene	2
TR V pseudogene	2
IG C pseudogene	1
scaRNA	1
scRNA	1
TR J gene	1

e

	Nucleus number	Reads per nucleus	Average genes	Average UMIs
snRandom-seq	1077	24597	3633	12135
DroNc -seq	5636	160000	3295	4643
sNucDrop -seq	1984	23000	2665	5195

Supplementary Figure 1. Establishment of cell encapsulation platform and high-sensitivity snRNA-seq data from a human-mouse mixture sample by snRandom-seq

a, Image of DAPI-stained 293T and 3T3 nuclei before cell encapsulation. Scale bar: 50 µm. **b**, Design of the device for cell, bead and mix reagents encapsulation. **c**, Species-mixing scatter plot shows the single-nuclei capture efficiency and doublet rate of snRandom-seq without pre-indexed random primers. **d**, Counts of biotypes detected by snRandom-seq in the 293T nuclei. **e**, Sensitivity comparison among snRandom-seq, DroNc-seq[1] and sNucDrop-seq[2].

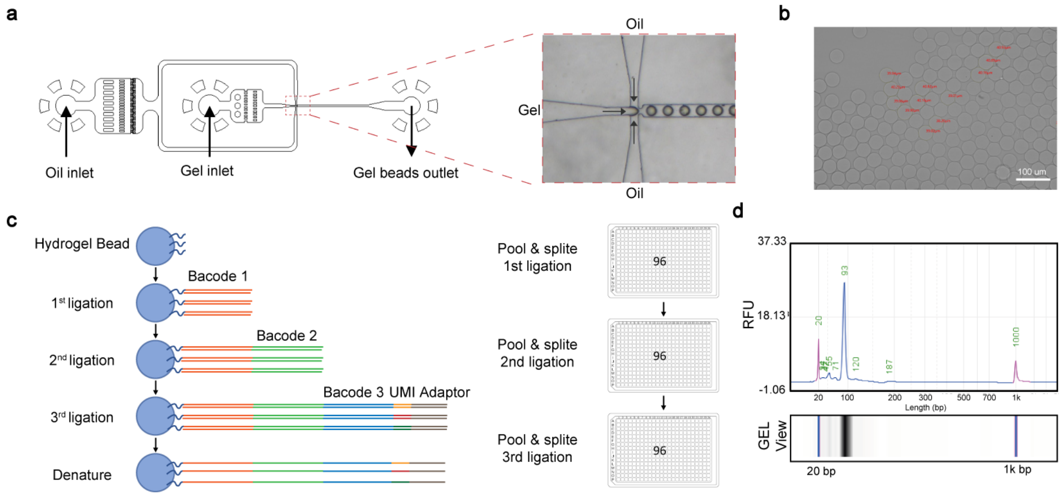

Supplementary Figure 2. Generation of barcode beads

a, Design of the device for hydrogel beads generation. **b**, Image of hydrogel beads. Scal bar: 100 µm. **c**, Overview of barcode synthesis process. **d**, Electropherogram of released primers for Qsep100™ DNA Fragment Analyzer. Lower marker: 20 bp; upper marker: 1k bp.

a

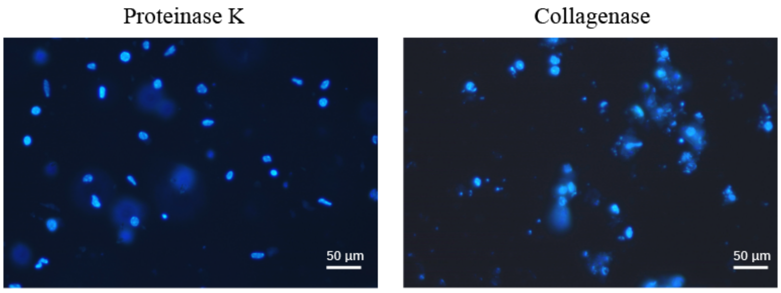

Supplementary Figure 3. Nuclei isolated from FFPE mouse kidney by Proteinase K and Collagenase.

a, DAPI images of nuclei isolated from FFPE mouse kidney by Proteinase K (0.5 mg/ml) and Collagenase (1.5 mg/ml). Scale bar, 50 µm.

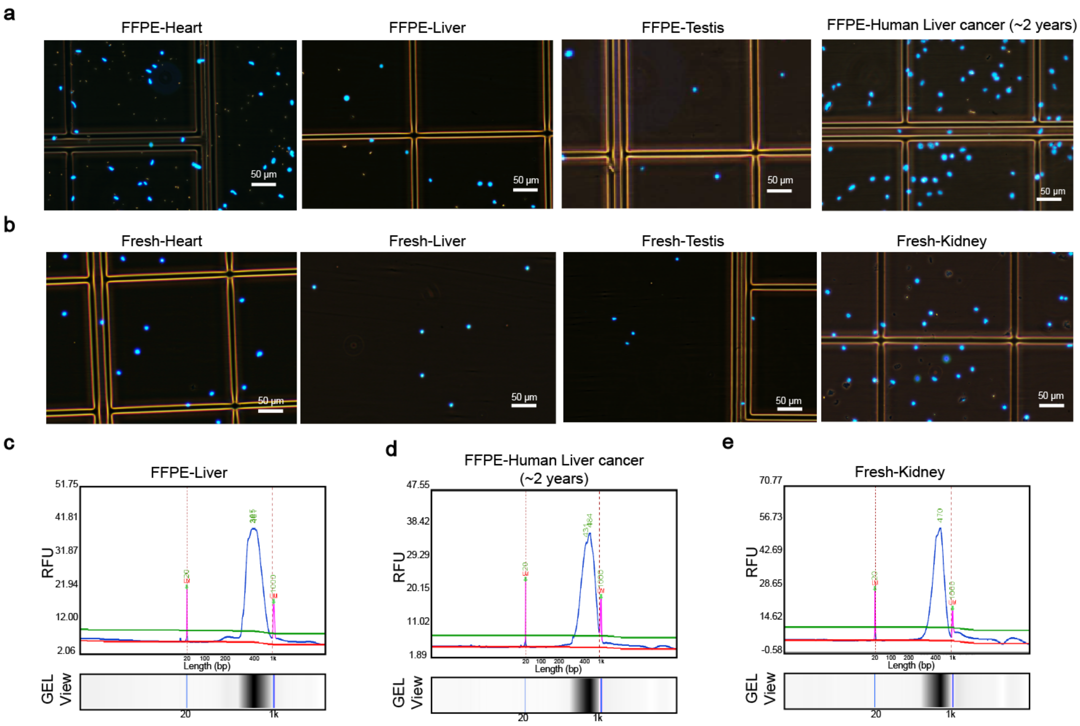

Supplementary Figure 4. Single nuclei and cDNA libraries generated by snRandom-seq from FFPE and fresh samples.

a, DAPI images of nuclei isolated from FFPE mouse tissues, including heart, liver, testis, and an about 2-year-old FFPE human liver cancer. Scale bar, 50 μm. **b**, DAPI images of nuclei isolated from fresh mouse tissues, including heart, liver, testis, kidney. Scale bar, 50 μm. **c-e**, Electropherogram of cDNA libraries from FFPE mouse liver (**c**), FFPE human liver cancer (**d**), and fresh mouse kidney(**e**). Lower marker: 20 bp; upper marker: 1k bp.

a

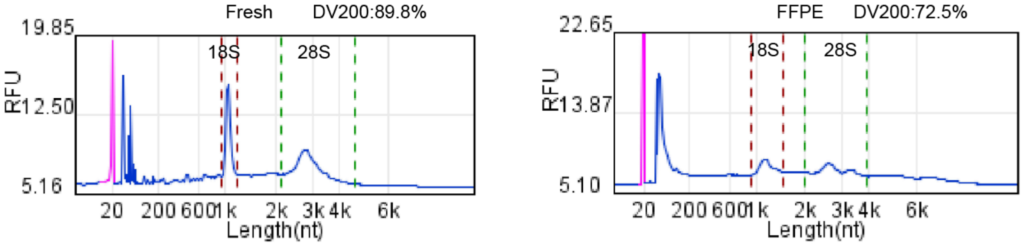

Supplementary Figure 5. Comparison of the RNA quality of FFPE and fresh samples.

a, Electropherogram of RNAs isolated from FFPE mouse testis and fresh mouse testis. DV200 value, the percentage of fragments >200 nucleotides.

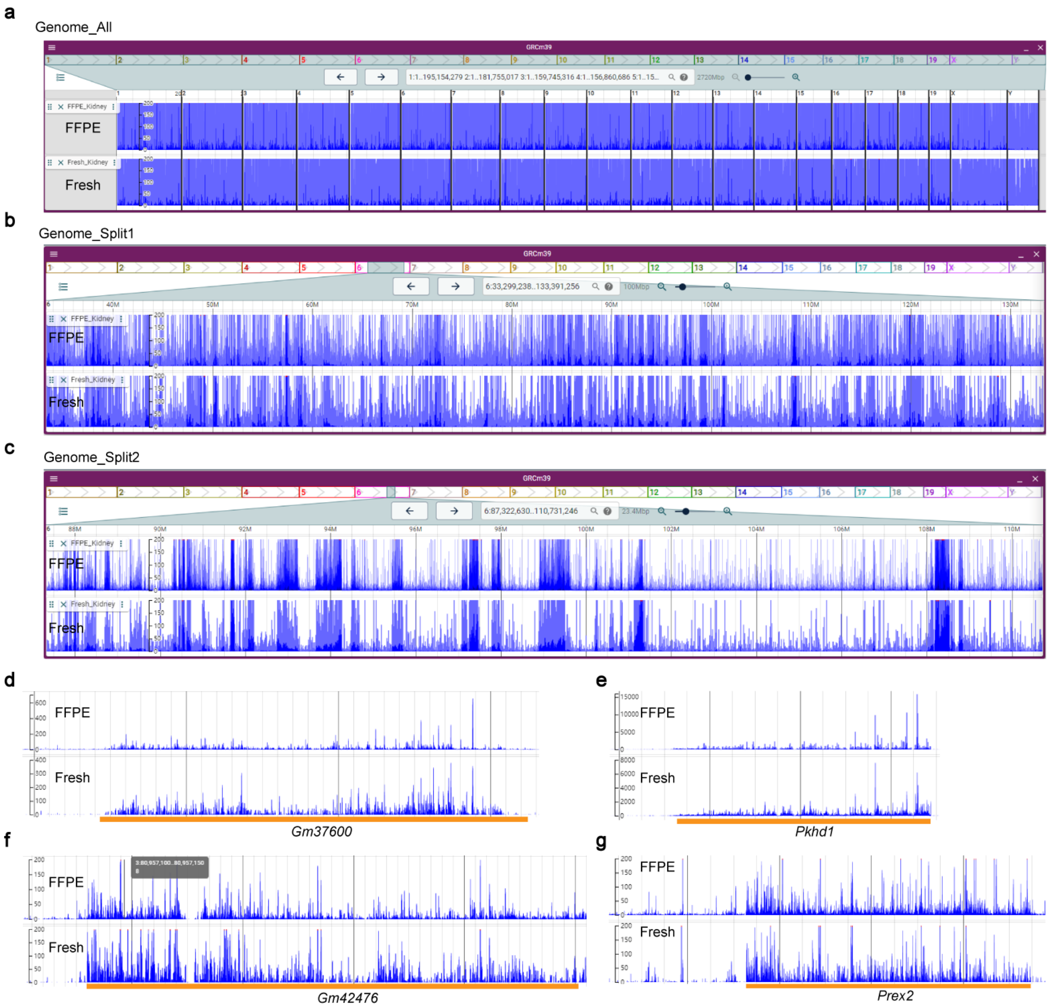

Supplementary Figure 6. Comparison of the genome coverage of FFPE and fresh single nuclei RNA profiles by snRandom-seq.

a-c, The whole (**a**) and split (**b**, **c**) merged genome browser tracks visualized by JBrowse. **d-g**, The reads distribution of four randomly selected genes (*Gm37600*, *Pkhd1*, *GM42476*, and *Prex2*) in the merged genome browser track visualized by JBrowse.

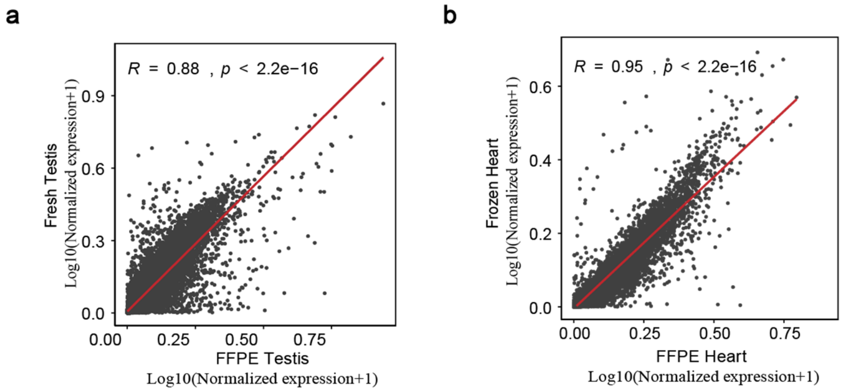

Supplementary Figure 7. Comparison of the gene expression of FFPE and fresh samples by snRandom-seq

a, **b**, The Pearson's correlation coefficient (*R*) of the normalized gene expressions between FFPE/fresh mouse testis samples (**a**) and mouse heart samples (**b**). Each dot represents the average expression level of a gene. The red line indicates the linear regression line. *p*-value (*p*) was computed from two-sided permutation test. FFPE testis nuclei: n= 3865. Fresh testis nuclei: n= 1795. FFPE heart nuclei: n=6942. Frozen heart nuclei: n= 10974.

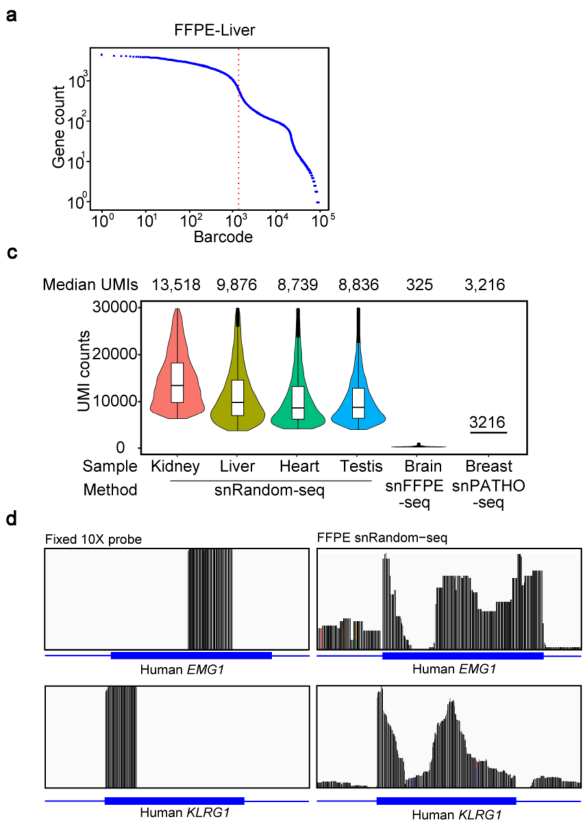

b

Biotype	snFFPE-seq	snRandom-seq
protein coding	12996	19564
lncRNA	921	7743
processed pseudogene	28	2259
TEC	9	918
unprocessed pseudogene	3	693
snRNA	NA	580
transcribed unprocessed pseu	7	180
transcribed processed pseudo	4	157
snoRNA	NA	71
polymorphic pseudogene	2	47
unitary pseudogene	1	44
miRNA	NA	38
pseudogene	1	29
rRNA	NA	27
transcribed unitary pseudoger	3	24
Mt tRNA	NA	19
TR V gene	NA	15
misc RNA	NA	14
IG V pseudogene	NA	12
IG C gene	NA	10
IG V gene	NA	10
TR C gene	NA	7
TR J gene	NA	4
ribozyme	NA	3
scaRNA	NA	3
translated unprocessed pseud	1	2
Mt rRNA	NA	2
TR V pseudogene	NA	2
IG C pseudogene	NA	1
IG J gene	NA	1
TR J pseudogene	NA	1

Supplementary Figure 8. snRNA-seq data from FFPE mouse samples by snRandom-seq

a, Barcode-gene count plot of FFPE mouse liver. Barcodes of FFPE mouse liver single nucleus were ordered from the largest to smallest gene counts. **b**, Counts of different biotypes detected in FFPE mouse liver by snRandom-seq and mouse brain by snFFPE-seq[3]. **c**, Distribution of UMI counts detected from different FFPE tissues, including mouse tissues (heart, kidney, testis and liver) and human liver cancer using snRandom-seq, comparing with the mouse brain using snFFPE-seq[3] and breast cancer using snPATHO-seq[4]. Kidney nuclei: *n*= 5,795, liver nuclei: *n*= 4,287, heart nuclei: *n*= 6,732, testis nuclei: *n*= 3,774, brain nuclei: *n*= 7,031, breast nuclei: *n*= 5,721. Data are presented as median values. Data in the box plot correspond to the first (lower hinges) quartiles, third quartiles (upper hinges), and median (center). The upper whisker extends from the hinge to the maxima no further than 1.5 * IQR from the hinge. The lower whisker extends from the hinge to the minima at most 1.5 * IQR of the hinge. **d**, Representative raw reads aligned to human genes (*EMG1*and *KLRG1*) generated by snRandom-seq and 10X Chromium Fixed RNA Profiling.

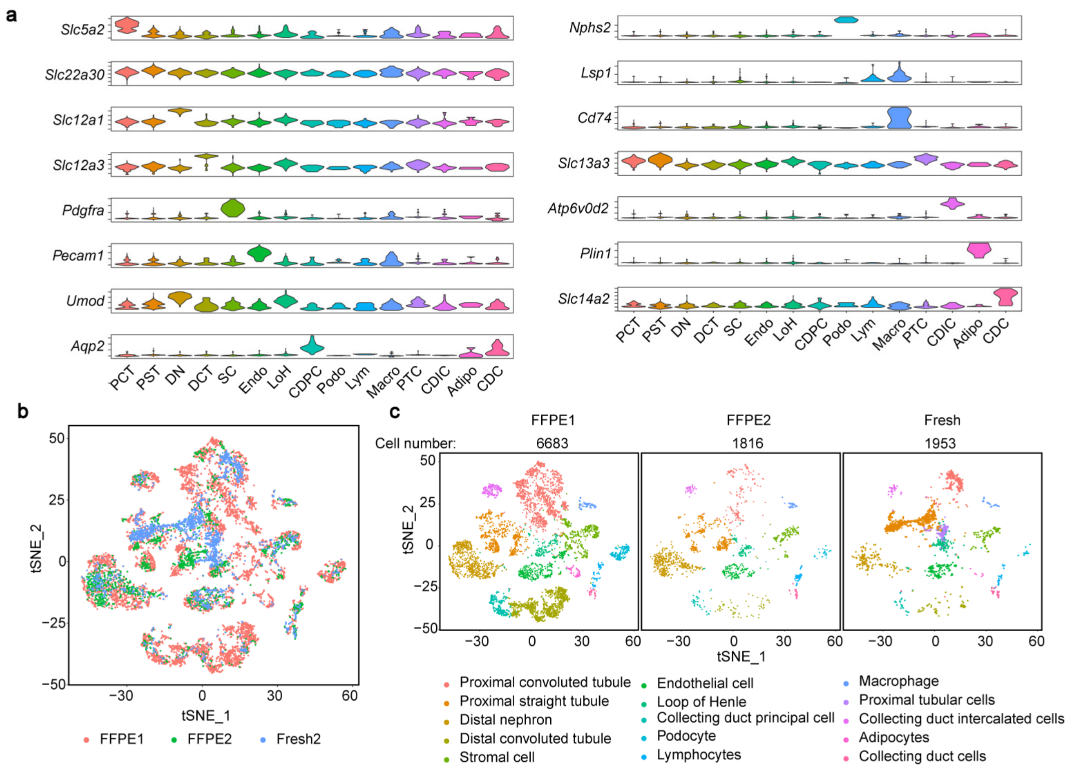

Supplementary Figure 9. Comparison of FFPE and fresh samples from the same tissue by snRandom-seq

a, Gene expressions of known marker genes for cell type identification. **b**, *t*SNE map of integrated snRandom-seq data of FFPE1, FFPE2 and fresh sample. Each colored point refers to a sample of the three kinds of samples. **c**, The integrated *t*SNE map contributed by snRandom-seq data from FFPE1, FFPE2 and fresh sample, respectively. Cell types annotated from the integrated snRandom-seq data shown below.

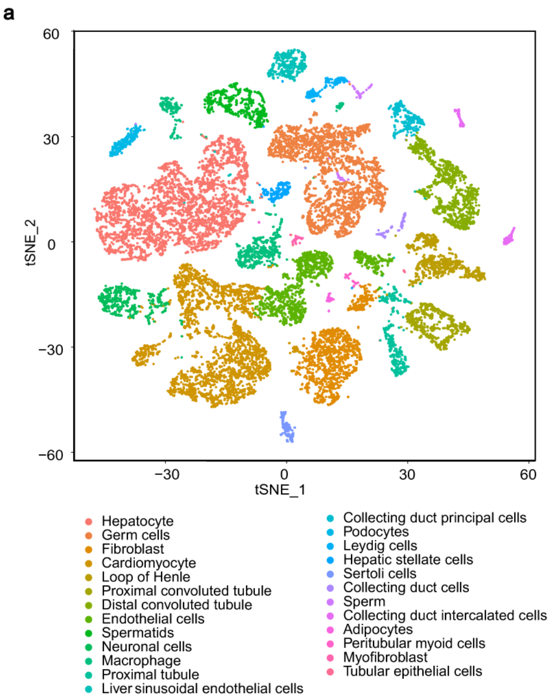

b

Cell type	Count	Marker gene
Hepatocyte	3460	*Alb*
Germ cells	2601	*Dazl*
Fibroblast	1433	*Pdgfra*
Cardiomyocyte	2928	*Tnnt2*
Loop of Henle	789	*Umod*
Proximal convoluted tubule	631	*Slc5a2*
Distal convoluted tubule	1088	*Slc12a3*
Endothelial cells	1405	*Pecam1*
Spermatids	615	*Tex29*
Neuronal cells	580	*Abcc9*
Macrophage	889	*Cd163/Cd68*
Proximal tubule	481	*Ace*
Liver sinusoidal endothelial cells	420	*Fcgr2b*
Collecting duct principal cells	327	*Aqp2*
Podocytes	257	*Cdkn1c*
Leydig cells	216	*Cyp11a1*
Hepatic stellate cells	211	*Reln*
Neurons	176	*Sema5b*
Collecting duct cells	162	*Slc14a2*
Sperm	119	*Tnp2*
Collecting duct intercalated cells	210	*Atp6v0d2*
Adipocytes	90	*Plin1*
Peritubular myoid cells	72	*Acta2*
Myofibroblast	70	*Msln*
Tubular epithelial cells	28	*Nos1*
Total	19258	

Supplementary Figure 10. Cell atlas identified by snRandom-seq in multiple different FFPE mouse tissues

a, *t*SNE map of integrated snRandom-seq data of FFPE mouse tissue sample (heart, liver, kidney and testis). Each colored point refers to different identified cell types. **b**, Cell numbers of different identified cell types and known marker genes.

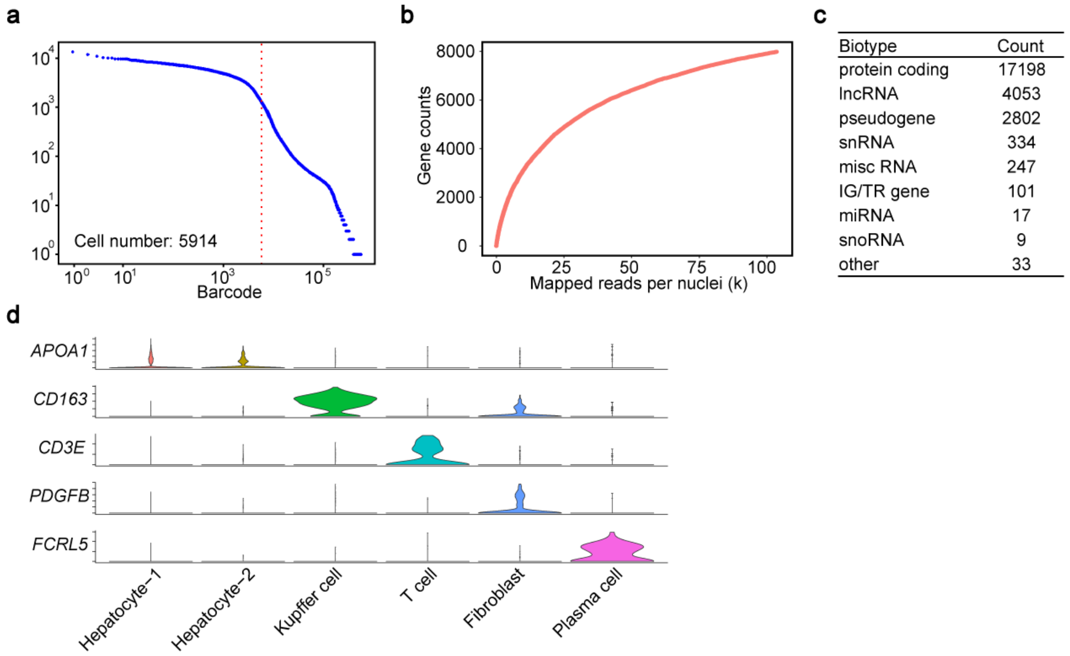

Biotype	Count
protein coding	17198
lncRNA	4053
pseudogene	2802
snRNA	334
misc RNA	247
IG/TR gene	101
miRNA	17
snoRNA	9
other	33

Supplementary Figure 11. snRandom-seq identified cell types in clinical FFPE sample

a, Barcode-gene count plot of FFPE human liver cancer sample. **b**, Saturation analysis of snRandom-seq based on the sample. **c**, Counts of different biotypes detected in the sample by snRandom-seq. **d**, Gene expressions of known marker genes for cell type identification.

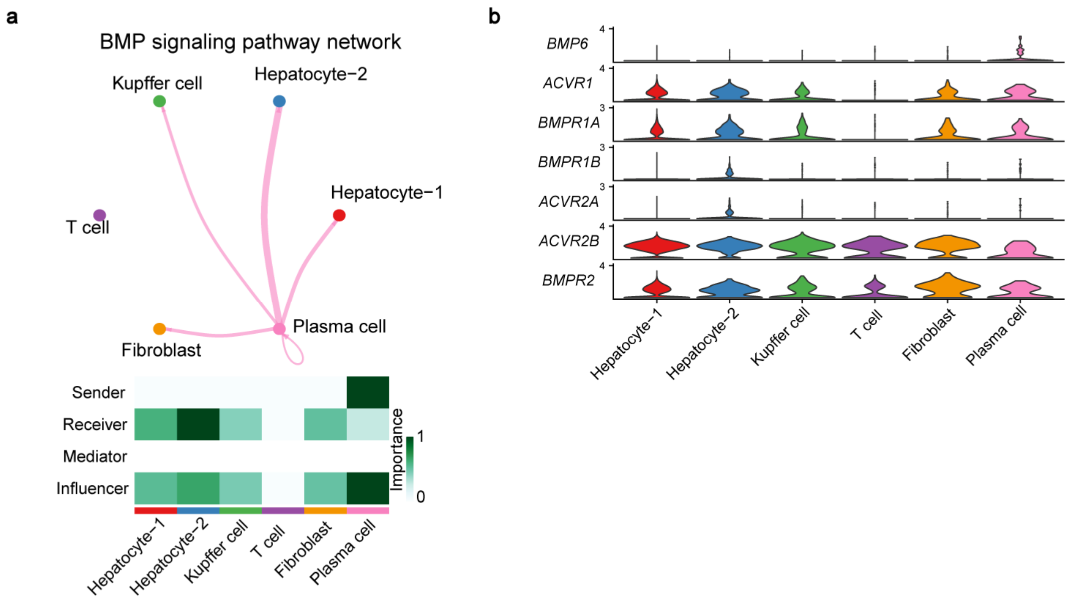

Supplementary Figure 12. snRandom-seq detected different receptor-ligand interactions in sub-populations

a, The inferred BMP signaling pathway network. Heatmap showing the relative importance of each cell cluster based on the computed four network centrality measures of BMP signaling network. **b**, Expression of BMP signaling network-related receptors and ligands.

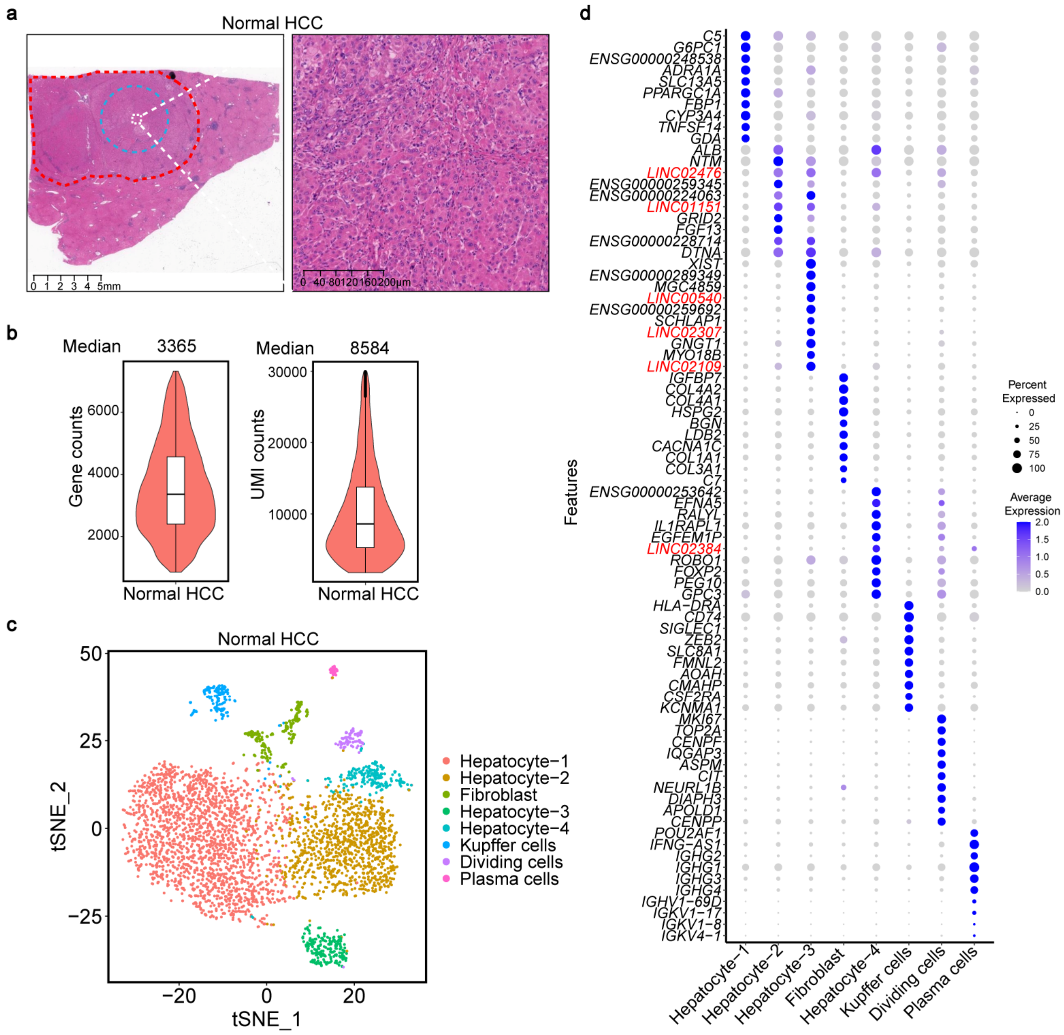

Supplementary Figure 13. snRandom-seq detected different lncRNAs in subpopulations of human normal HCC FFPE specimens.

a, Histological appearance of normal HCC at low magnification (left, Scale bar, 5 mm.) and high magnification (right, Scale bar, 200 μm). Red broken line: tumorous area. Blue broken line: sampling area. White broken line: magnified area. **b**, Violin plots and box plots showing the number of genes and UMIs detected in normal HCC FFPE specimens by snRandom-seq. Normal HCC nuclei: *n*= 6046. Data are presented as median values. Data in the box plot correspond to the first and third quartiles (lower and upper hinges) and median (center). Data in the box plot correspond to the first (lower hinges) quartiles, third quartiles (upper hinges), and median (center). The upper whisker extends from the hinge to the maxima no further than 1.5 * IQR from the hinge. The lower whisker extends from the hinge to

the minima at most 1.5 * IQR of the hinge. **c**, t-SNE map of nuclei isolated from the FFPE sample based on their gene expressions. Eight cell types, including four hepatocellular subtypes, were annotated and shown. **d**, Top ten markers of each of the eight cell types. Red font: lncRNAs.

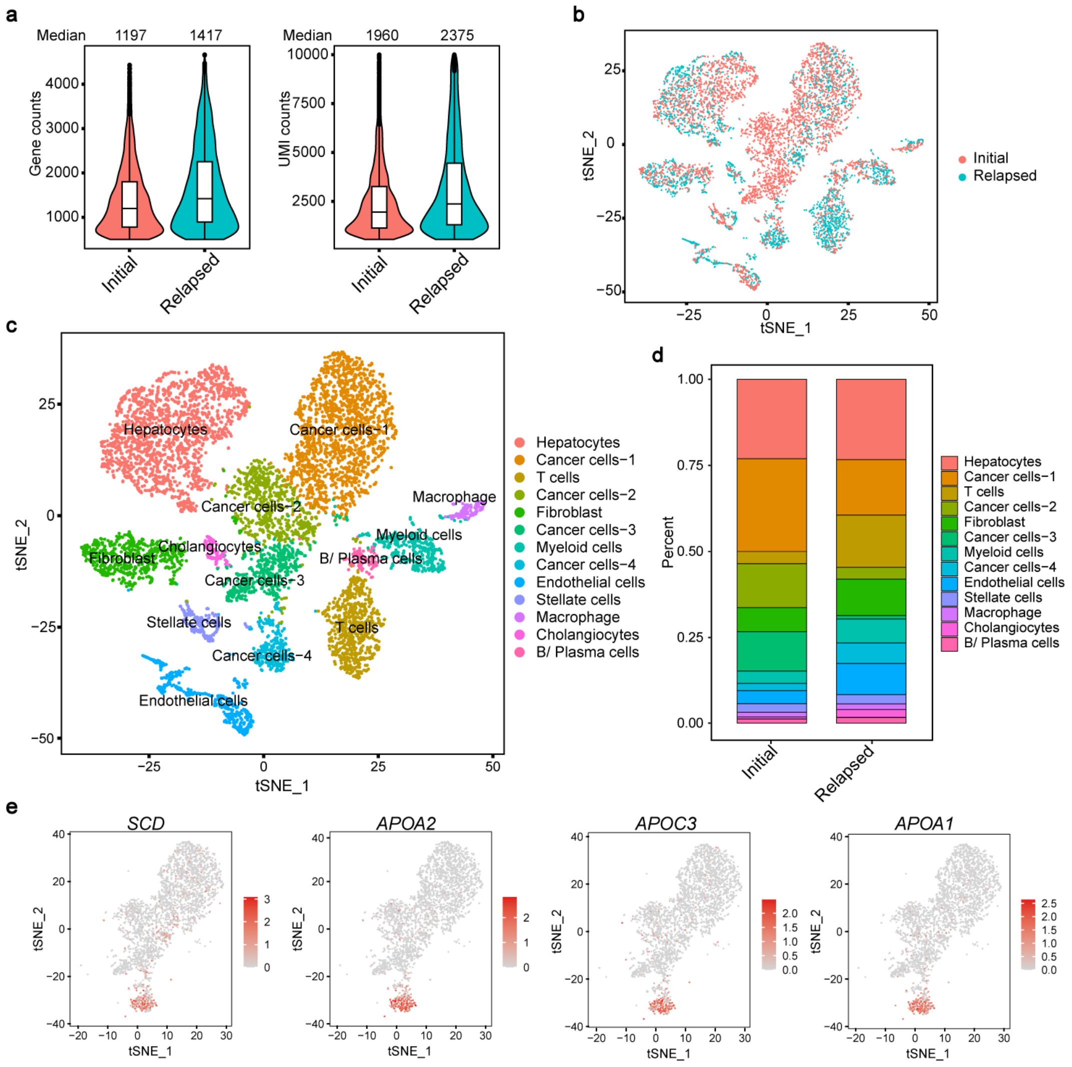

Supplementary Figure 14. snRandom-seq revealed cell heterogeneity of initial and relapsed FFPE specimens.

a, Violin plots and box plots showing the number of genes and UMIs detected in initial and relapsed FFPE specimens from a same colorectal cancer liver metastasis patient (CRLM) by snRandom-seq. Initial CRLM nuclei: n= 4556. Relapsed CRLM nuclei: n= 3587. Data are presented as median values. Data in the box plot correspond to the first (lower hinges) quartiles, third quartiles (upper hinges), and median (center). The upper whisker extends from the hinge to the maxima no further than 1.5 * IQR from the hinge. The lower whisker extends from the hinge to the minima at most 1.5 * IQR of the hinge. **b-c**, *t*-SNE map of integrated nuclei from initial and relapsed FFPE specimens colored by groups (initial,

relapsed) (**b**) and cell types (hepatocytes, cancer cells, T cells, fibroblasts, myeloid cells, endothelial cells, stellate cells, macrophages, cholangiocytes, B/ plasma cells) (**c**). **d**, Percent of cells in the different cell types. **e**, Expression levels of lipid metabolism related genes (*SCD, APOA2, APOC3,* and *APOA1*) in cancer cells.

Supplementary Tables

Supplementary Table 1: Primers

**Primers list**	**Sequence**
Block primer	GAGAATGTGAGTGAAGATGTATGGTGANNNNNNN
Random primer 1	GGAGTTGGAGTGAGTGGATGAGTGATGGAAGGAATNNNNNNN
Random primer 2	GGAGTTGGAGTGAGTGGATGAGTGATGGATGAATGNNNNNNN
Random primer 3	GGAGTTGGAGTGAGTGGATGAGTGATGGATATGTGNNNNNNN
Random primer 4	GGAGTTGGAGTGAGTGGATGAGTGATGGTGGTGTANNNNNNN
Random primer 5	GGAGTTGGAGTGAGTGGATGAGTGATGGTAGGTTANNNNNNN
oligo(dT) primer 1	GGAGTTGGAGTGAGTGGATGAGTGATGGAAGGAATTTTTTTTTTTTTTT
oligo(dT) primer 2	GGAGTTGGAGTGAGTGGATGAGTGATGGATGAATGTTTTTTTTTTTTTT
oligo(dT) primer 3	GGAGTTGGAGTGAGTGGATGAGTGATGGATATGTGTTTTTTTTTTTTTT
oligo(dT) primer 4	GGAGTTGGAGTGAGTGGATGAGTGATGGTGGTGTATTTTTTTTTTTTTT
oligo(dT) primer 5	GGAGTTGGAGTGAGTGGATGAGTGATGGTAGGTTATTTTTTTTTTTTTT
Barcoded beads primer	/5Acryd/ATTATATATAT U GTG AGT GAT GGT TGA GGA TGT GTG GAGATA [10 bases barcode1] TGGT [10 bases barcode2] GAGA [10 bases barcode3] NNNNNNNNTTTTTTTTTTTTTTTTTTTT
PCR primer 1	GGAGTTGGAGTGAGTGGATGAGTGATG
PCR primer 2	GTG AGT GAT GGT TGA GGA TGT GTG GAG ATA

Supplementary Table 1: Primers

List of primers used in snRandom-seq. Block primer was used for DNA blocking. Random primer 1-5 and oligo(dT) primer 1-5 were used for reverse transcription. Barcoded beads primer was the structure of the primers crosslinking with beads. The sequences of barcode1, barcode2, and barcode3 were provided in Supplementary Table 2. PCR primer 1 and PCR primer 2 were used for PCR amplification.

Supplementary Table 2: Barcode sequences

Barcode 1	Barcode 2	Barcode 3
GATGTACATG	ATAGAAACGA	GCTTTTCTTG
TTCAGATTCT	TCCGTGCAAG	CTTTAAGCAT
CGTACTACGC	AATGGGACAC	GAAATATTGT
GCTATTATAG	TATTAGGCGA	GGCGGAAACG
CAGAAGGAAC	CAATCATCAC	AATGGGTATG
AGACTTTTAA	CAGTTTTACT	CGGCAATTGA
GGCAACTGTG	CTTTCTAGAT	CTCGAGGAAT
AAATTCGCGG	TGTGATACCT	CCTATTGTCT
CTTAGACACT	TGTTAACAAC	GAGGCTCCTA
AGCGGACTAT	TTGCTTTCAG	ACTCAAGGGA
GAGACGCTAC	CAGGAACTAA	ACATCCTGCT
GTCATCGTGC	TCGGGCGGAT	ATTATGGTAG
TCATTAACAG	ACGTATAGTG	TACCGGATTA
GCTTCGCTTA	TACTTAAGGC	CAATTTGAAC
CTGGCAAAGA	CATGCGGGTG	TTTATAGACC
TCGCTTGAAG	TCGCACTCCG	ATCACTCGGT
ATGAACTTTG	GAAACTGAAA	ACTACTAATA
CAGAGATACG	TACAGGCGCA	CCGGAGGATA
GGCTGATAAA	GTTGGCATTT	GACGGGAGGT
TATTTGTCGC	GTTTTAGGAA	TCTGGAATAG
TTAAGGCAAA	AGATTGGCGG	TGGAGGGAGC
ACAGTATTAT	GAGCGGGTTT	GCTTGGCAAC
TGGATGCTAT	CGGGAAAGGG	GAGGAATCCT
TCGAATTTCG	TTCTTAGGCG	AAGTAAACGG
TACTATGGCG	AACACAATAA	TTTTGTGGTG
TAGCTTAGTG	TTCCGGCGAG	TCATTGATTG
GTCAGGCATG	TTTGTTTTAT	CAGTATCATC
GGTTGGTGGT	GCAGGTGCAG	CACATGGTGT
ACATGTAGCT	ATAAGGACCT	GTATAGAGTG
GCGAACAATG	TCAGTTATCC	TCGGGTAATC
TGTTGCTTGC	AATGGAATGT	AATTAAGAGC
TAACAGCAAT	TGTAATTGAT	TCACAGCACA
GTTTGGACTC	TCAGTGGGTT	AATTACCTCT
CATTATGCTA	GTGGACGTTG	TGTTCATAGA
GAGTAAGTGA	CTCCTGCAAA	CACGGTGTAG
GAGCGGTGTA	TTCGGATTCC	GCCGAGGTGT
CGTAAGTCGA	AATCGGGTCA	ATGAGGCTTT

TGGCACGTTT	AGTCGCCGAC	TGATGGCGGA
GAAATCTTTT	CATAAGAAGT	CGCTCCTGGT
AACAACGCAA	AGGCAGGAAA	TCAAGGCCTG
ATGTTGTGGC	CTTATCCTTG	ATTAAAGGCT
TAACTCATTC	GGTCGAAGGG	CGGAAAGAGT
TCGACTATAT	CAAGAGGTGA	ACAATAGGCG
TATCAGTCTG	CTACTGGTGG	TCCGACCGTC
CTATATGTAG	TATGTTCATC	GAAGATACAA
TGCAATACGG	AGGCTTGTTC	CTAAAGGACG
AAACTGGTGT	TCGACAGCAG	TATAAAGGAA
AATTAGGCGT	GTAAATGGGA	ACAGGAGTGA
ACTGTTCAGG		
CAGTATTGTT		
GTACTATAGT		
ACAGACATTT		
GTCTTTGGAT		
CTTGATCTAT		
GTATGTCAGG		
TATTCAACTG		
TTTTGACGGA		
ATGGTAGAAA		
GTGCTCTATC		
GTTATAGTTG		
AGGATCTTAC		
TGCATTTGAA		
AATTCGGATA		
GGTTGAGAGC		
CAAGGCTCAT		
GAATGGAAGC		
CTTCTATATG		
TCTGGATTAC		
TTGATCAGTA		
ATTTTTGTCG		
TACGACTGAA		
AAAGTTGCAA		
GGCGTGCTTT		
CGGCGGATTT		
CGACAAAGTG		
TGCGTAATTC		
CAAATGTCGT		

CAGCTAGCTT		
ACACGGCTCT		
ATTATGACTG		
ACGCATAGAC		
TGGAAGGACT		
TAAGTAGATA		
CACAAGTATA		
TAAGAATGCG		
ATATGTGTGA		
ACACAGTGTG		
AGCACTGTAT		
ACTATGCGAG		
CTACATGCTC		
CTTCTGGTGG		
GGATGCTCGA		
CAGCGAAGGA		
GTAAAGACGT		
TTGCTACAAC		
TGGAAACGGT		

Supplementary Table 2: Barcode sequences

Barcode 1, 2 and 3 were used for the three rounds of split-and-pool-based ligation in the barcoded beads synthesis of snRandom-seq (**Supplementary Fig. 2c**).

Supplementary Notes

Supplementary Note 1: snRandom-seq Protocol 1.0

Ziye Xu, Wang Lab

Department of Laboratory Medicine, the First Affiliated Hospital,

Zhejiang University School of Medicine

1. Single nuclei isolation from FFPE samples

Dewax and rehydrate the FFPE samples using the following program:

Xylene I 5min

Xylene II 5min

100% ethanol I 3min

100% ethanol II 3min

95% ethanol 1min

90% ethanol 1min

85% ethanol 1min

80% ethanol 1min

70% ethanol 1min

50% ethanol 1min

30% ethanol 1min

Wash the dewaxed and rehydrated samples twice with 1 mL of pre-cold wash buffer.

FFPE nuclei lysis buffer:

10% Triton X-100 20 μL

RNase Inhibitor (200 U/μL) 1 μL

1X PBS 2 mL

Homogenize the dewaxed and rehydrated samples with Dounce homogenizer (working volume: 2 mL) with the presence of 1 mL of pre-cold FFPE nuclei lysis buffer.

10 mg/mL proteinase K:

proteinase K 100 mg

ddH_2O 10 mL

After homogenization, add 1 mL of FFPE nuclei lysis buffer to rinse the homogenizer, then add 100 μL of 10 mg/mL proteinase K into the lysis buffer and put on PCR block 37ºC for 5 min, then quickly put

on ice.

Filter the isolated nuclei through a 20-μm cell strainer and washed twice with wash buffer.

2. *In situ* DNA block

Count the FFPE nuclei under the microscope, then dilute with 1X PBS. A total of 100,000~1000,000 FFPE nuclei were used for *in situ* DNA blocking. The DNA Polymerase kit was ordered from M20 Genomics.

Make the following *in situ* DNA block reaction mix:

Nuclei (100,000~1000,000) in 1X PBS 25.5 μL

10 μM block primer 5 μL

DNA Polymerase 2 μL

5X DNA polymerization buffer 10 μL

100 mM dNTP 5 μL

RNase Inhibitor (200 U/μL) 2.5 μL

Mix well and then put on PCR block 37ºC for 30 min, then quickly put on ice.

PBST buffer:

T-ween 20 5 μL

RNase Inhibitor (200 U/μL) 5 μL

1X PBS 10 mL

Wash the nuclei with 1 mL of PBST buffer three times, then resuspend with 1X PBS.

3. *In situ* reverse transcription

The reverse transcription kit was ordered from M20 Genomics.

Make the following *in situ* reverse transcription reaction mix:

Nuclei (100,000~1000,000) in 1X PBS 22.5 μL

10 μM random primer 5 μL

10 μM oligo(dT) primer 5 μL

Reverse Transcriptase 2.5 μL

5X reverse transcription buffer 10 μL

100 mM dNTP 2.5 μL

RNase Inhibitor (200 U/μL) 2.5 μL

Mix well and then put on PCR block to run following program:

12 cycles of
8ºC 10s
15ºC 15s
20ºC 15s
30ºC 30s
42ºC 1min
End cycles
42ºC 30min
4 ºC Forever

Wash the nuclei with 1 mL of PBST buffer three times, then resuspend with 1X PBS.

4. dA tailing

The TdT reaction kit was ordered from NEB.
Prepare the following dA tailing reaction mix:
Nuclei (100,000~1000,000) in 1X PBS 39 µL
10X TdT reaction buffer 5 µL
10 X $CoCl_2$ 5 µL
TdT enzyme (20,000 U/mL) 0.5 µL

Mix well and then put on PCR block 37ºC for 30 min, wash with 1 mL of PBST three times, then resuspend with 1X PBS.

5. Droplet barcoding

Count the nuclei under microscope, then dilute with a 30% density gradient solution at 1000 nuclei/uL. The 2X DNA extension reaction mix was ordered from M20 Genomics. The barcoded beads were customized with M20 Genomics company.

Encapsulate nuclei (40 uL), 2X DNA extension reaction mix (40 uL) and barcoded beads (10 uL) into droplets using the microfluidic platform. Then, split the emulsions into 2 PCR tubes and then put on PCR block to run following program:
37ºC 1hour
50ºC 30min
60ºC 30min
75ºC 20min

4 ºC Forever

After the barcoding reaction, mix the droplets with PFO buffer. Take out the aqueous phase and then use Ampure XP beads to purify DNA fragments. Elute the purified DNA fragments with 40 uL ddH$_2$O.

Prepare the following PCR amplification reaction mix:
10X PCR reaction buffer 5 μL
10 uM Primer1 1 μL
10 uM Primer2 1 μL
cDNA Template 35~40 μL
Add ddH$_2$O to total 50 μL

Mix well and then put on PCR block to run following program:
95ºC 1min
20 cycles of
95 ºC 30s
60 ºC 30s
72 ºC 2min
End cycles
72 ºC 5min
4 ºC Forever

Use Ampure XP beads to purify the PCR products.

6. Library preparation

After amplification and purification, VAHTS Universal DNA Library Prep Kit for Illumina V3 was used to construct library. Library sequencing was performed using the NovaSeq 6000 and S4 Reagent Kit with paired end reads of 150.

Supplementary References

1. Habib, N. et al. Massively parallel single-nucleus RNA-seq with DroNc-seq. *Nat. Methods* **14**, 955-958 (2017).
2. Hu, P. et al. Dissecting Cell-Type Composition and Activity-Dependent Transcriptional State in Mammalian Brains by Massively Parallel Single-Nucleus RNA-Seq. *Mol. Cell* **68**, 1006-1015.e1007 (2017).
3. Chung, H. et al. SnFFPE-Seq: towards scalable single nucleus RNA-Seq of formalin-fixed paraffin-embedded (FFPE) tissue. *bioRxiv*, 2022.2008.2025.505257 (2022).
4. Vallejo, A.F. et al. snPATHO-seq: unlocking the FFPE archives for single nucleus RNA profiling. *bioRxiv*, 2022.2008.2023.505054 (2022).
