# Supplementary material for: High-throughput single nucleus total RNA sequencing of formalin-fixed paraffin-embedded tissues by snRandom-seq
Source: Nat Commun. 2023 May 12;14:2734. doi: 10.1038/s41467-023-38409-5 (PMC10182092; doi:10.1038/s41467-023-38409-5)
Supplement: Supplementary file 3 — Reporting Summary [file 41467_2023_38409_MOESM3_ESM.pdf]

nature portfolio

Corresponding author(s): Dr. Yongcheng Wang, Dr. Guoji Guo, Dr Longjiang Fan

Last updated by author(s): Apr 24, 2023

Reporting Summary

Nature Portfolio wishes to improve the reproducibility of the work that we publish. This form provides structure for consistency and transparency in reporting. For further information on Nature Portfolio policies, see our Editorial Policies and the Editorial Policy Checklist.

Statistics

For all statistical analyses, confirm that the following items are present in the figure legend, table legend, main text, or Methods section.

n/a	Confirmed	
☐	☒	The exact sample size (*n*) for each experimental group/condition, given as a discrete number and unit of measurement
☐	☒	A statement on whether measurements were taken from distinct samples or whether the same sample was measured repeatedly
☐	☒	The statistical test(s) used AND whether they are one- or two-sided<br>*Only common tests should be described solely by name; describe more complex techniques in the Methods section.*
☒	☐	A description of all covariates tested
☐	☒	A description of any assumptions or corrections, such as tests of normality and adjustment for multiple comparisons
☐	☒	A full description of the statistical parameters including central tendency (e.g. means) or other basic estimates (e.g. regression coefficient) AND variation (e.g. standard deviation) or associated estimates of uncertainty (e.g. confidence intervals)
☐	☒	For null hypothesis testing, the test statistic (e.g. *F*, *t*, *r*) with confidence intervals, effect sizes, degrees of freedom and *P* value noted<br>*Give P values as exact values whenever suitable.*
☒	☐	For Bayesian analysis, information on the choice of priors and Markov chain Monte Carlo settings
☒	☐	For hierarchical and complex designs, identification of the appropriate level for tests and full reporting of outcomes
☐	☒	Estimates of effect sizes (e.g. Cohen's *d*, Pearson's *r*), indicating how they were calculated

Our web collection on statistics for biologists contains articles on many of the points above.

Software and code

Policy information about availability of computer code

Data collection	BGIMAGING CellView (x64, 4.11.20068.20211225) was used to acquire images.<br>Q-Analyzer for Qsep100 (3.4.3.0.6593) was used to acquire images of gel views of DNA fragments.
Data analysis	1. STARsolo in STAR (2.7.10a) was used to perform genome mapping, barcode sorting and UMI counting.<br>2. Seurat (v3 & v4.1.1) in R (4.2.1) was used to process count matrix, and do downstream analysis (UMAP, Differential gene expression, Cell cycle).<br>3. scvelo (0.2.4) and velocyto.R (0.6) was used to perform RNA velocity analysis.<br>4. bedtools (2.26.0) and IGV(2.13.2) was used to calculate transcriptome coverage and generate genome coverage plot.<br>5. ggpubr (0.4.0) in R(4.2.1) was used to do correlation analysis.<br>6. ggplot2 (3.3.5) in R(4.2.1) was used to generate raw plots.<br>7. Adobe Illustrator (22.1) was used to typeset.<br>8. AutoCAD (2021) was used to design microfluidic devices.<br>9. Q-Analyzer for Qsep100 (3.4.3.0.6593) was used to analyze DNA fragments.

For manuscripts utilizing custom algorithms or software that are central to the research but not yet described in published literature, software must be made available to editors and reviewers. We strongly encourage code deposition in a community repository (e.g. GitHub). See the Nature Portfolio guidelines for submitting code & software for further information.

Data

Policy information about availability of data

All manuscripts must include a data availability statement. This statement should provide the following information, where applicable:

- Accession codes, unique identifiers, or web links for publicly available datasets
- A description of any restrictions on data availability
- For clinical datasets or third party data, please ensure that the statement adheres to our policy

The snRanThe snRandom-seq snRNA-seq datasets generated in this study have been deposited in the Genome Sequence Archive under accession code CRA010745 (293T-3T3 mixture and mouse FFPE tissues) [https://ngdc.cncb.ac.cn/gsa-human/browse/CRA010745] and HRA003712 (MTM-HCC and normal HCC FFPE samples) [https://ngdc.cncb.ac.cn/gsa-human/browse/HRA003712]. The public scRNA-seq data of 293T and 3T3 cell mixture by 10X Chromium Single Cell 3' Solution V3 used in this study are available in the Short Read Archive under accession code SRP073767 [https://www.ncbi.nlm.nih.gov/sra/?term=SRP073767]. The public scRNA-seq data of 293T cells by VASA-drop is available at the Gene Expression Omnibus under accession code GSE176588 [https://www.ncbi.nlm.nih.gov/geo/query/acc.cgi?acc=GSE176588]. Source data are provided with this paper.

Human research participants

Policy information about studies involving human research participants and Sex and Gender in Research.

Reporting on sex and gender	FFPE tissues of clinical human cancers were collected from male patients.
Population characteristics	1. FFPE tissues of macrotrabecular-massive hepatocellular carcinoma (MTM-HCC) were collected from a 43, male, Chinese patient by surgical resection.<br>2. FFPE tissues of normal HCC were collected from a 45, male, Chinese patient by surgical resection.<br>3. FFPE tissues of initial and relapsed colorectal cancer liver metastasis (CRLM) were collected from a 64, male, Chinese patient by biopsy (initial age 62, relapsed age 64).
Recruitment	Patients with pathological diagnosis of MTM-HCC, normal HCC or relapsed CRLM were recruited randomly. Clinical information was collected after writing informed consents. There are no self-selection bias or any bias that may be present.
Ethics oversight	The collection of human samples and research conducted in this study were approved by the Research Ethics Committee of the First Affiliated Hospital, Zhejiang University School of Medicine (approval numbers: IIT20220893A).

Note that full information on the approval of the study protocol must also be provided in the manuscript.

Field-specific reporting

Please select the one below that is the best fit for your research. If you are not sure, read the appropriate sections before making your selection.

☒ Life sciences ☐ Behavioural & social sciences ☐ Ecological, evolutionary & environmental sciences

For a reference copy of the document with all sections, see nature.com/documents/nr-reporting-summary-flat.pdf

Life sciences study design

All studies must disclose on these points even when the disclosure is negative.

Sample size	No sample size calculation was performed. Sample size was determined to be adequate based on the magnitude and consistency of measurable differences between groups.
Data exclusions	No data were excluded from analysis.
Replication	The microfluidic encapsulation experiment, FFPE single nuclei isolation experiment, beads synthesis experiment, DNA fragments analysis experiment, proteinase K and collagenase comparison experiment, and RNA quality (DV200) comparison experiment were repeated more than five times independently with similar results. The 293T-3T3 mixture experiment and DNA block experiment were repeated three times independently with similar results.
Randomization	Half of each mouse tissue was randomly selected and made into FFPE samples. The other half of each mouse tissue was immediately used. The FFPE tissues of clinical human cancer were collected from tissues with obvious pathological diagnosis under the supervision of professional pathologist.
Blinding	Blinding was not included in the study because no bias could be made by the subject or the tester in the experiments performed.

Reporting for specific materials, systems and methods

We require information from authors about some types of materials, experimental systems and methods used in many studies. Here, indicate whether each material, system or method listed is relevant to your study. If you are not sure if a list item applies to your research, read the appropriate section before selecting a response.

Materials & experimental systems

n/a	Involved in the study
☒	☐ Antibodies
☐	☒ Eukaryotic cell lines
☒	☐ Palaeontology and archaeology
☐	☒ Animals and other organisms
☒	☐ Clinical data
☒	☐ Dual use research of concern

Methods

n/a	Involved in the study
☒	☐ ChIP-seq
☒	☐ Flow cytometry
☒	☐ MRI-based neuroimaging

Eukaryotic cell lines

Policy information about cell lines and Sex and Gender in Research

Cell line source(s)	HEK293T cells (human, ATCC CRL-3216) and 3T3 cells (mouse, ATCC CL-173) were ordered from company (Procell Life Science&Technology).
Authentication	None of the cell lines have been authenticated.
Mycoplasma contamination	Cell lines were not tested for mycoplasma contamination but no indication of contamination was observed.
Commonly misidentified lines (See ICLAC register)	None of the cell lines used in this study was found in the database of misidentified cell lines.

Animals and other research organisms

Policy information about studies involving animals; ARRIVE guidelines recommended for reporting animal research, and Sex and Gender in Research

Laboratory animals	Male wildtype C57BL6/J mice (6~8 weeks of age) were ordered from Shanghai SLAC Laboratory Animal. Mice were single housed under standard laboratory conditions, including a 12 h light/dark cycle, temperatures of 18-23°C with 40-60% humidity, with free access to mouse diet and water.
Wild animals	No wild animals were used in the study.
Reporting on sex	Only male mice were used in the study. Sex was determined based on similar studies in this field.
Field-collected samples	No field collected samples were used in the study.
Ethics oversight	All the procedures involving mice were approved by Zhejiang University Animal Care and Use Committee (approval numbers: ZJU20170466).

Note that full information on the approval of the study protocol must also be provided in the manuscript.
